# Impact of media trust and personal epidemic experience on epidemic prevention behaviors in the context of COVID-19: A cross-sectional study based on protection motivation theory

**DOI:** 10.3389/fpubh.2023.1137692

**Published:** 2023-04-13

**Authors:** Dan Zhang, Fan Su, Xiaoxia Meng, Zhixin Zhang

**Affiliations:** ^1^School of Medicine and Health Management, Guizhou Medical University, Guiyang, China; ^2^Pharmaceutical Economic Management Research Center, Guizhou Medical University, Guiyang, China; ^3^Guizhou Institute of Health Development, Guizhou Medical University, Guiyang, China; ^4^College of Humanities and Management, Guizhou University of Traditional Chinese Medicine, Guiyang, China; ^5^School of Accounting, Dianchi College of Yunnan University, Kunming, China

**Keywords:** protection motivation theory, media trust, personal epidemic experience, COVID-19, PLS-SEM

## Abstract

**Objective:**

This study aimed to elucidate the impact of media trust on epidemic prevention motivation and behaviors based on the Protection Motivation Theory (PMT) and to evaluate the moderation effect of personal epidemic experience, which focused on the differences in two groups with or without epidemic experience.

**Methods:**

The exogenous constructs and PMT model and scale were constructed through literature analysis, and a web-based questionnaire survey was conducted among 428 individuals aged above 18 years in China. Statistical analysis and hypothesis testing were performed in SPSS 26 and SmartPLS 3.

**Results:**

Traditional media trust accounted for the largest weight in media trust (w = 0.492, *p*-value < 0.001), followed by social media (w = 0.463, *p*-value < 0.001), and interpersonal communication (w = 0.290, *p*-value < 0.001). Media trust was positively and significantly related to both threat appraisal (β = 0.210, *p*-value < 0.001) and coping appraisal (β = 0.260, *p*-value < 0.001). Threat appraisal (β = 0.105, *p*-value < 0.05) and coping appraisal (β = 0.545, *p*-value < 0.001) were positively and significantly related to epidemic prevention motivation, which positively and significantly related to epidemic prevention behaviors (β = 0.492, *p*-value < 0.001). The R^2^ values of epidemic prevention motivation and behavior are 0.350 and 0.240, respectively, indicating an acceptable explanation. Multiple-group analysis revealed five significant differences in paths between the two groups, indicating personal epidemic experience acting as a slight moderator on these paths.

**Conclusion:**

Traditional media trust and social media trust were the important elements in COVID-19 prevention and control, and public health departments and governments should ensure the accuracy and reliability of information from traditional and social media. Simultaneously, the media should balance threat information and efficacy information in order to generate the public’s prevention motivation and behaviors.

## 1. Introduction

The Corona Virus Disease 2019 (COVID-19) outbreak has become a global public health crisis that is seriously endangering human life and health and causing disorder in politics, economy, education, business, transportation, social life, entertainment, and the provision of health services all over the world (1–12). To limit the spread of the virus, most nations implemented pharmaceutical and interventions to control the epidemic, including vaccination rollouts, travel bans, university and school closures, the enforcement of face masking, social distancing, and quarantine or lockdowns (4, 13–17). Moreover, fighting the epidemic requires a concerted effort from both the government and individuals. Regarding the individuals, epidemic protective behaviors, such as mask wearing, hand washing, and social distancing, have been approved as essential for preventing and slowing the spread of COVID-19 (18). However, the epidemic has lasted longer than expected. The emergence of new highly-virulent variants, such as the delta and omicron variants, emphasizes the fact that it is impossible to eradicate the virus completely, while it is likely to transit from pandemic to endemic (19). This indicated that people may need to be prepared to deal with localized and seasonal outbreaks in the future (20). At the early stage of the epidemic, the public regularly practiced good protective habits, but over time, the recurrence of the epidemic resulted in pandemic prevention fatigue, thus affecting their compliance with the recommended health precautions to protect themselves (21) and their daily habits and health behaviors, including waking up, going to bed for sleep, and even using the Internet (4, 22). Therefore, it is essential to encourage the public to continuously practice health preventive behaviors to protect themselves from COVID-19. Thus, exploring the influence factors and their impact on preventive behaviors implemented by the public during the epidemic is of vital importance. Many scholars have used different health behavior theories to explain the underlying reasons for people’s preventive motivation and behaviors to prevent the epidemic, such as the health belief model (HBM) (23, 24), theory of planned behavior (TPB) (18, 25–27), protection motivation theory (PMT), and others. The PMT is the most common of these. PMT is a developed form of the health belief model, first proposed by Rogers (28) and revised in 1983 (29). The threat appraisal (including perceived severity, perceived vulnerability, intrinsic and extrinsic rewards) and coping appraisal (including response efficacy, self-efficacy, response costs) pathways explain the willingness or unwillingness to adopt a health behavior. Of these, intrinsic and extrinsic rewards are factors that weaken threat appraisal, and response costs are factors that weaken coping appraisal (29). Currently, PMT has been used to explain a wide range of behaviors and can be applied to various populations and contexts, especially in public health emergencies such as SARS, HINI, and H7N9.

In the past 3 years, there have been several studies on health behaviors related to COVID-19. Some researchers only discussed the intention or motivation of health behaviors without discussing the specific health behaviors (30, 31), whereas others discussed various health protective behaviors in the context of COVID-19, such as hand washing, disinfection, social distance, and other behaviors, as shown in Supplementary Table S1 [references (32–36) are cited in Supplementary material]. All of them discussed several or a list of behaviors without categorizing them.

Although many researchers have proposed different exogenous variables, such as trust in the government (37), knowledge of COVID-19 (36, 38, 39), past behavior (25), and COVID-19 experiences (26, 40), several others focused on the media. Li and Sun proposed media trust, including trust in traditional media, social media, and interpersonal communication, as an exogenous variable that influences motivation to receive vaccinations for COVID-19 (41). Adiyoso chose media use (including print, radio, TV, WhatsApp, social media, domestic web, and overseas web) to explain social distancing intentions (27). In fact, when people use the media they trust, it can effectively result in changes in behaviors. Additionally, since the COVID-19 pandemic has lasted for a long time, people have different COVID-19 experiences and different prior exposures to COVID-19. It could be assumed that people who have experienced or have not experienced an epidemic may have a moderate effect on their epidemic prevention motivation and behaviors. Based on the above theory and research, we sought to answer the following questions:

Q1: What is the relationship between public media trust, PMT constructs, and health prevention behaviors in a public health emergency?

Q2: During the COVID-19 crisis, do people with different personal epidemic experiences have differences in forming health protective behaviors?

## 2. Literature review and hypotheses

### 2.1. Research framework

**Figure 1:**
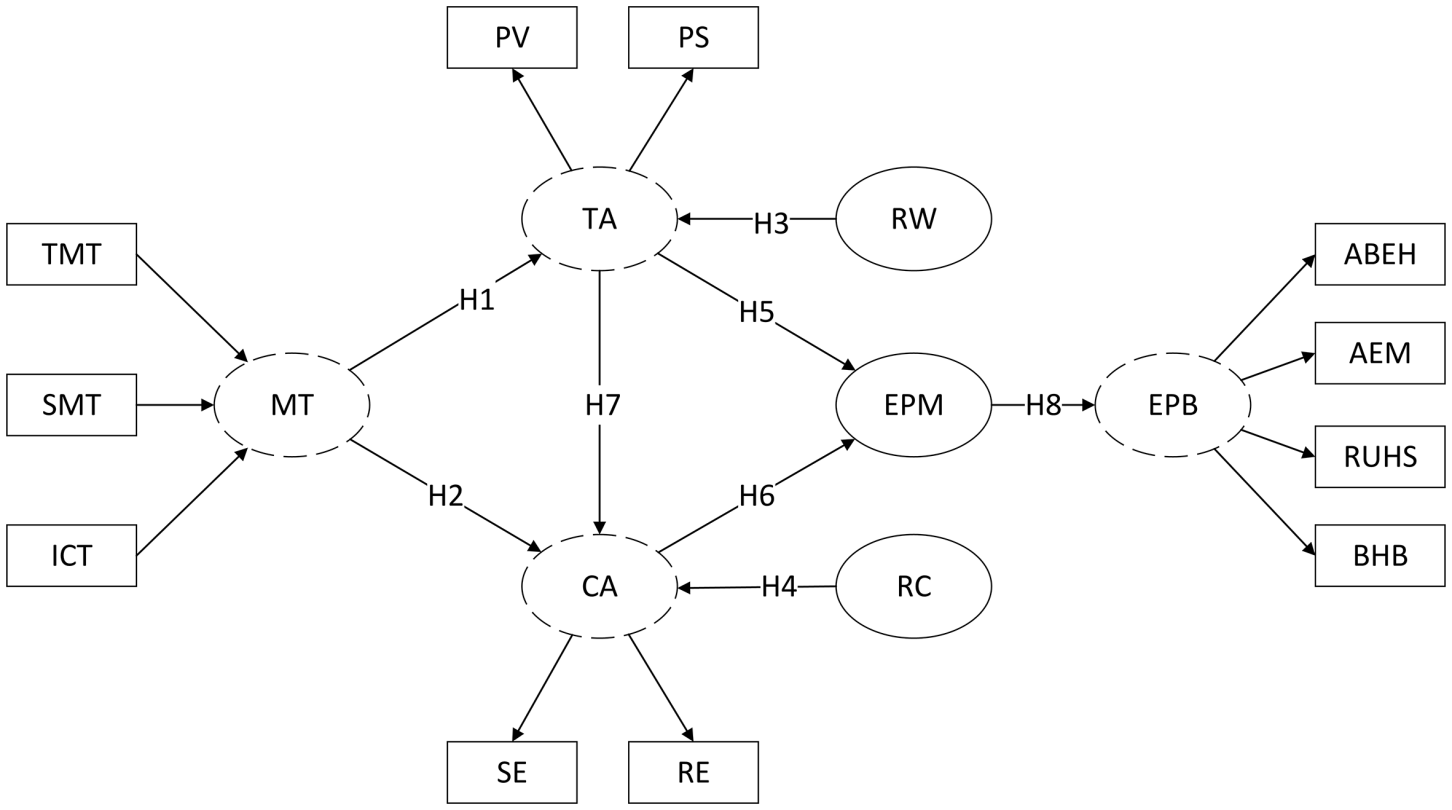
Research model. 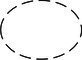 Second-order construct. TMT, Traditional Media Trust; SMT, Social Media Trust; ICT, Interpersonal Communication Trust; MT, Media Trust; TA, Threat Appraisal; CA, Coping Appraisal; PV, Perceived Vulnerability; PS, Perceived Severity; SE, Self-efficacy; RE, Response Efficacy; RW, Rewards; RC, Response Costs; EPM, Epidemic Prevention Motivation; EPB, Epidemic Prevention Behaviors; ABEH, Avoidance Behaviors of Environmental Hazards; AEM, Anti-epidemic Measures; RUHS, Rational Use of Health Services; BHB, Basic Health Behaviors.

Figure 1 shows the proposed research model with all the theoretical constructs of the current study. Unlike most previous studies that only utilized PMT variables, including threat appraisal (TA), coping appraisal (CA), perceived vulnerability (PV), perceived severity (PS), self-efficacy (SE), response efficacy (RE), rewards (RW), response costs (RC), epidemic prevention motivation (EPM), and epidemic prevention behaviors (EPB) (33), this study integrated the PMT variables and exogenous variables of media trust (MT), which consist of traditional media trust (TMT), social media trust (SMT), and interpersonal communication trust (ICT). In addition, epidemic prevention behaviors (EPB) were further subdivided into four constructs, including avoidance behavior of environmental hazards (ABEH), anti-epidemic measures (AEM), rational use of health services (RUHS), and basic health behaviors (BHB). Notably, MT, TA, CA, and EPB are second-order constructs; therefore, there is no hypothetical path to their sub-constructs separately. To handle these hierarchical component models, we chose the extended repeated indicators approach, which assigned all the lower-order components to higher-order components (42).

### 2.2. Development of hypotheses

According to the model of behavior formation, the theoretical framework of PMT is divided into three parts: information source, cognitive mediation process, and response mode. As a result, the source of information is the starting point for protection motivation (43). Individuals actually obtain information through various media channels, which is the source of information. With the development of media technology, media communication has deeply influenced individuals’ cognition, attitude, motivation, and behavior in relation to health, thus becoming one of the most important methods for health intervention. In particular, media coverage is an important source of risk information (44). People rely more on media for their information during an epidemic, increasing the demand for epidemic information and the exchange of opinions. Epidemic information on media can magnify people’s fear and urge them take preventive behaviors (45). In fact, it is the information obtained from their trusted media that can stimulates the public’s threat appraisal (TA), form the subjective cognition of risks, and guide individual behaviors to some extent (41). Moreover, access to a large amount of information leads to a more comprehensive understanding of the outbreak and a clear understanding of preventive measures, thus facilitating the public’s coping appraisal (CA) of the crisis.

Therefore, media trust (MT) is one of the essential factors affecting health behavior, thus acting as the exogenous variable of PMT. Media trust can be divided into traditional media trust (TMT), social media trust (SMT), and interpersonal communication trust (ICT) (41), which are all necessary parts of the MT construct. Therefore, according to the view of Fornell (46), MT is a second-order formative construct because it is a combination of TMT, SMT, and ICT, all of which are causes of the MT construct.

Thus, this study proposed the following hypotheses.

*H1:* Media trust (MT) is positively related to threat appraisal (TA).

*H2:* Media trust (MT) is positively related to coping appraisal (CA).

The PMT contains threat appraisal and coping appraisal, which form the core of the cognitive process and contribute to the individual’s motivation or intention to adopt protective behaviors (28). Threat appraisal assesses the level of threat perceived by the individual and contains sub-constructs such as perceived vulnerability, perceived severity, and rewards. Perceived susceptibility assesses the likelihood of a person being affected by a disease. Perceived severity assesses how serious an individual perceives the threat. Rogers’ (1983) revision of PMT includes the rewards for not taking the recommended response, which is also part of the threat appraisal process. The higher the rewards for not taking the response, the less likely an individual is to take it. Since rewards are negatively related to threat appraisal, while vulnerability and severity are positively related, we can define threat appraisal as a second-order construct, and vulnerability and severity as sub-constructs of it, except for rewards. Therefore, the greater an individual’s perceived vulnerability and severity of threat, the greater the perceived threat, the lower the rewards for not responding, the more motivated individuals are to protect themselves, and the more likely they are to form protective behaviors (47).

Coping appraisal is an individual’s assessment of the recommended measures, and it contains sub-constructs such as response efficacy, self-efficacy, and response cost. Response efficacy refers to beliefs about whether the recommended measures are effective in reducing the threat. Self-efficacy refers to individuals’ beliefs about whether they have the capacity to implement the recommended measures. Response costs refer to beliefs about how costly it will be for an individual to implement the recommendations, and the higher the response costs of adopting the response, the less likely the individual will adopt it. Since the response costs are negatively related to coping appraisal, while response efficacy and self-efficacy are positively related to it, we can define coping appraisal as a second-order construct and response efficacy and self-efficacy as sub-constructs to it, except for the response costs. Therefore, the greater an individual’s response efficacy and self-efficacy to countermeasures, the greater the coping appraisal, the lesser the response costs of countermeasures, the more motivated individuals are to protect themselves, and the more likely they are to adopt protective behaviors (47).

Some scholars believe that there is a certain sequence in individuals’ cognitive assessment processes. When a health threat event occurs, individuals first assess the severity and vulnerability of the event through acquired knowledge, thus forming a threat appraisal, then assess the countermeasures they can take (i.e., self-efficacy and response efficacy) through past experiences, subsequently forming a coping appraisal. Finally, through the interaction of threat appraisal and coping appraisal, a definite protective motivation will be generated, which will lead to protective behavior (48).

Previous research used health behavior theory to explain various types of health protective behaviors during the COVID-19 pandemic, such as hand washing, limiting social contact, disinfection of articles, wearing a mask, physical activity, and injection of the COVID-19 vaccine. Previous research measured health protective behaviors as a single behavior or as a simple list of protective behaviors. In fact, we can classify the health behaviors adopted during COVID-19, thus creating some new sub-constructs. According to the classification of health behaviors, we can categorize the health behaviors under four constructs: epidemics as avoidance behavior of environmental hazards (ABEH), anti-epidemic measures (AEM), rational use of health services (RUHS), and basic health behaviors (BHB). Thus, this study proposed the following hypotheses.

*H3:* Rewards (RW) are negatively related to threat appraisal (TA).

*H4:* Response costs (RC) are negatively related to coping appraisal (CA).

*H5:* Threat appraisal (TA) is positively related to epidemic prevention motivation (EPM).

*H6:* Coping appraisal (CA) is positively related to epidemic prevention motivation (EPM).

*H7:* Threat appraisal (TA) is positively related to coping appraisal (CA).

*H8:* Epidemic prevention motivation (EPM) is positively related to epidemic prevention behaviors (EPB).

As the epidemic continues and recurrences occur in China, some areas have experienced one or more rounds of the COVID-19 outbreak, while other areas have not, which has led to some people having direct experience with COVID-19, whereas others do not. Previous studies have shown that just like “ripple,” people who are closer to the disaster have higher levels of emotion and perceived risk (49), as well as more avoidance behavior (50), implying that the current environment and past experiences may influence behavioral decisions (41). Furthermore, people with and without personal epidemic experience have different levels of risk perception and anxiety, which affect their protective motivation and behavior. Thus, personal epidemic experience should be taken into consideration. As a result, this study further proposed the following hypothesis.

*H9:* The TPB model has a significant difference between the personal epidemic experience group and the no epidemic experience group.

## 3. Materials and methods

### 3.1. Research design

The current research is a cross-sectional study that aims to explore and validate the model. It takes the protection motivation theory (PMT) as the theoretical framework and the public’s media trust as a second-order formative external variable to explore the relationship between media trust and PMT variables, thus constructing a second-order structural equation model. It also explores whether having a personal experience with an epidemic has an effect on the model-that is, whether the model paths are significantly different.

The research design of the current study was based on variance-based structural equation modeling. Structural equation modeling (SEM) is prevalent in social sciences and has two main techniques, namely covariance-based SEM and variance-based SEM. The former is a traditional structural equation model, which is a multivariate statistical technique combining regression analysis, factor analysis, and ANOVA. The latter uses the Partial Least Squares Method (PLS), a mathematical optimization technique combining multiple linear regression analysis, typical correlation analysis, principal component analysis, and causal modeling. PLS, according to Ringle et al., has a number of advantages, including the ability to accept small samples and non-normally distributed data, as well as analyze formative constructs and handle complex models for exploratory studies and theory development (51). The statistical method used in this study is the partial least squares structural equation modeling (PLS-SEM) method, not the traditional CB-SEM method, since the latter requires a larger sample size and normally distributed variable data, which is more unsuitable for the validation of complex models. PLS-SEM, on the other hand, necessitates a smaller sample size, does not require normally distributed data, and has distinct advantages in the validation of second-order complex models and the processing of formative conformations (52).

### 3.2. Participants

Considering the risk of COVID-19 transmission, this study conducted an online survey for the public on the Tencent questionnaire platform from 1 March 2022, until 7 March 2022, when China implemented zero-COVID policy and strategies. The link to the survey was sent to different WeChat groups. Participation was open to anyone residing in China with a self-reported age of over 18 years old. The introduction of the questionnaire provided an informed consent form that explained the purpose of the study, the potential risks and benefits of participating in the study, the confidentiality measures for personal privacy, and the voluntary nature of participation in the survey. Participants first read the informed consent form and then began to fill out the questionnaire if they agreed. Participants could stop filling out the questionnaire at any time during the process. The complete completion of the questionnaire was considered consent to participate in this survey. Otherwise, it was considered unwilling to participate in the research. Finally, a total of 450 questionnaires were collected, excluding those with too many inconsistent options, and 428 valid questionnaires were retained, with an effective response rate of 95.1%. The Supplementary Dataset S1 was included in the Supplementary material. The ethics review of this study was approved by Dianchi College of Yunnan University, with approval letter No. 024-2022.

### 3.3. Measures

To ensure content validity, the measurement items were modified from relevant existing literature and translated into Chinese. Several scholars discussed and revised it several times. Then, a pretest was conducted to modify the instruments. The complete questionnaire consisted of 48 items for 14 first-order constructs and 4 s-order constructs with reflective–formative type and reflective–reflective type questions (53), as shown in Supplementary Table S2 [references (33, 35–37, 41) are cited in the Supplementary material]. A five-point Likert scale was used for all question items. Items of TMT, SMT, and ICT were scored using a Likert Scale of 1 (very low) to 5 (very high). Other items were measured on a Likert scale of 1 (strongly disagree) to 5 (strongly agree).

Personal epidemic experience was identified by a multiple-choice question that asked respondents, “Since the outbreak of COVID-19, which of the following situations have you now or ever experienced?” The response options were as follows: (1) My health code is yellow; (2) My health code is red; (3) My neighborhood has been blocked; (4) The area where I live used to be a medium or high-risk area; (5) Someone I know around me has been diagnosed with COVID-19; (6) I have been diagnosed with COVID-19; (7) I was quarantined in the hotel; (8) I was asked to be home-based quarantined; and (9) none of the above. The respondents who chose option nine were classified as the no epidemic experience group. The others who selected one or more options from 1 to 8 were classified as being in the epidemic experience group.

### 3.4. Data analysis

#### 3.4.1. Descriptive statistics

The data collected on the Tencent questionnaire platform were imported into SPSS 26.0 for data cleaning, and 428 samples were finally retained. SPSS 26.0 was also used for descriptive statistics analysis by obtaining the frequency of socio-demographic variables, such as gender, age, education, and epidemic experience.

#### 3.4.2. PLS-SEM

This study applied PLS to analyze the outer model and the inner model, and most of the examinations in this study followed the two-step approach put forward by Anderson and Gerbing with the software of SmartPLS 3.0. The first step is outer model analysis, including reliability and validity testing, while the second step is inner model analysis, including estimating and validating the structural model’s path coefficients and explanatory power. The preceding two steps are intended to confirm whether the constructs are reliable and valid, thereby validating the relationships between constructs (54, 55).

In the first step, the validity test includes a convergent validity test and a discriminant validity test. The convergent validity test was used to determine the degree of similarity of different measures in measuring the same concept. In this study, standardized factor loading of each indicator, reliability of each construct, and average variance extracted (AVE) of each construct were used to test convergent validity. Specifically, reliability was assessed using the composite reliability (CR), Cronbach’s alpha coefficients, or Dijkstra-Henseler’s rho. If any of the three values is greater than 0.6 or 0.7, the reliability is deemed desirable (55–57). According to Fornell and Larcker, a construct has good convergent validity if the standardized factor loadings of each indicator are greater than 0.6 or 0.7, the reliability values of each construct are greater than 0.6 or 0.7, and the average variance extracted (AVE) indicators of each construct are greater than 0.5 (58). The discriminant validity refers to the strength of the correlation coefficient among the latent constructs (59). The discriminant validity test was used to check whether a very high correlation existed between constructs in this study. There are several methods to identify the discriminant validity, one of them being the comparison of cross loading and factor loading for each indicator. When an item’s factor loading for its assigned latent construct is greater than its loading for any other construct, it indicates that it has reasonable discriminant validity (60). Besides, the heterotrait–monotrait (HTMT) ratio of correlations put forward by Henseler et al. was used here to evaluate discriminant validity. If the values of HTMT are lower than 0.9, the discriminant validity is favorable (61).

In the second step, path coefficients were used to represent the intensity and direction of the variable relationships to show the cause and effect between latent variables. Bootstrapping was used to estimate the significance (α = 0.05) of each path coefficient. Additionally, explanatory power was estimated by the R square value, which refers to the percentage to which the dependent variable can be explained, representing the predictive ability of the model (55). The value of R square lies between 0 and 1, and the larger the value, the stronger the explanatory power (60).

Finally, multiple-group analysis (MGA) was used to test the moderation effect of personal epidemic experience in order to determine if there were differences in all hypotheses between the epidemic experience group and the no epidemic experience group (62, 63). To examine the specific group differences by Multi Group Analysis (MGA), it is necessary to first establish the partial measurement invariance (64). SmartPLS provides a permutation algorithm to examine the measurement invariance of composite models (MICOM), which contains three steps, namely configural invariance, compositional invariance, and scalar invariance. Step 1 is configural invariance testing, which is established automatically but not displayed by SmartPLS 3. Step 2 is to test compositional invariance, which can check the invariance of the measurement path index weights between two groups. Step 3 is to test scalar invariance to examine whether the initial responses to the scale items differ between two groups. If the permutation *p*-values of both steps 2 and 3 are not significant, then full measurement invariance exists in the model. However, if only the permutation p-values in step 2 are not significant, the model has partial measurement invariance, and MGA should be used to compare the groups (65).

## 4. Results

### 4.1. Descriptive statistics analysis

Among the respondents, 182 (42.5%) were male and 246 (57.5%) were female; 26 (6.1%) were aged between 18 and 24 years old, 144 (33.6%) were aged between 25 and 30 years old, 153 (35.7%) were aged between 31 and 40 years old, 47 (11%) were aged between 41 and 50 years old, and 58 (13.6%) were over 50 years old; 34 (7.9%) were in junior high school and below, 67 (15.7%) in high school or technical secondary school, 268 (62.6%) were undergraduates or in college, and 59 (13.8%) were postgraduate students and above. Moreover, 198 (46.3%) had personal epidemic experience, while 230 (53.7%) had no personal epidemic experience.

### 4.2. Outer model analysis

A formative model was assessed by examining variance inflation factor (VIF) scores and the significance of the weights (63). In our study, since the MT construct was a second-order formative construct, it was necessary to assess its collinearity. The VIFs of the TMT, SMT, and ICT constructs were 1.308, 1.813, and 1.512, respectively, which were all less than the threshold value of 10, indicating that the collinearity assessment of the MT construct had not reached a significant level (66). Therefore, the collinearity did not adversely affect the path coefficient estimation of the structural model.

In the first step of PLS, the standardized factor loading and T-value of each item, the composite reliability, Cronbach’s alpha, Dijkstra-Henseler’s rho, and AVE of each construct were tested for convergent validity. As is shown in Table 1, since the MT construct is a second-order formative construct, it has no composite reliability, AVE, or Cronbach’s alpha, whereas the value of rho A is 1. The weights of its subordinate TMT, SMT, and ICT constructs are 0.492, 0.463, and 0.290, respectively, with T-values all > 1.96 and significant. Moreover, since there is only one measurement item for EPM, the values of standardized factor loading, the composite reliability, AVE, Cronbach alpha, and rho-A are 1, and the T-value is 0. Additionally, the standardized factor loadings of the measurement items of other constructs are significant (t > 1.96), between 0.711 and 0.948, greater than 0.7; the values of composite reliability of other constructs are between 0.868 and 0.938, greater than 0.7; the values of Cronbach’s alpha are between 0.774 and 0.939, greater than 0.7; the values of rho-A are between 0.778 and 0.942, >0.7; and the values of average extraction variance (AVE) are between 0.623 and 0.862, >0.5. Therefore, the convergent validity of the study was satisfactory (67).

**Table 1:** Reliability and convergent validity.

Construct	Items	Factor loading/weight	T-value	Composite reliability	AVE	Cronbach’s *α*	*rho-A*
MT (second-order formative construct)	TMT	0.492	19.333	-	-	-	1
	SMT	0.463	28.690				
	ICT	0.290	16.713				
TMT	TMT1	0.865	43.100	0.893	0.736	0.821	0.826
	TMT2	0.870	58.022				
	TMT3	0.838	42.242				
SMT	SMT1	0.769	24.429	0.878	0.706	0.790	0.799
	SMT2	0.893	74.755				
	SMT3	0.854	50.543				
ICT	ICT1	0.921	81.684	0.916	0.846	0.817	0.817
	ICT2	0.918	78.004				
TA (second-order reflective construct)	PV	0.864	51.263	0.884	0.792	0.845	0.847
	PS	0.915	89.127				
PV	PV1	0.841	45.971	0.871	0.692	0.777	0.778
	PV2	0.845	47.968				
	PV3	0.810	31.195				
PS	PS1	0.781	23.182	0.868	0.623	0.798	0.802
	PS2	0.815	30.094				
	PS3	0.830	32.753				
	PS4	0.728	21.555				
RW	RW1	0.727	3.489	0.901	0.646	0.879	1.031
	RW2	0.791	3.870				
	RW3	0.816	3.964				
	RW4	0.780	3.649				
	RW5	0.896	3.592				
CA (second order reflective construct)	RE	0.908	83.913	0.926	0.862	0.887	0.890
	SE	0.948	132.034				
RE	RE1	0.820	27.118	0.890	0.729	0.813	0.820
	RE2	0.904	79.508				
	RE3	0.836	34.638				
SE	SE1	0.796	30.910	0.888	0.666	0.832	0.835
	SE2	0.872	58.084				
	SE3	0.826	36.093				
	SE4	0.768	23.886				
RC	RC1	0.774	16.682	0.877	0.705	0.795	0.864
	RC2	0.910	55.644				
	RC3	0.830	25.617				
EPM	EPM 1	1	0.000	1	1	1	1
EPB (second-order reflective construct)	ABEH	0.764	20.474	0.915	0.729	0.939	0.942
	AEM	0.932	119.658				
	RUHS	0.834	42.595				
	BHB	0.876	72.493				
ABEH	ABEH1	0.788	28.439	0.887	0.664	0.829	0.843
	ABEH2	0.887	58.271				
	ABEH3	0.712	19.621				
	ABEH4	0.862	60.870				
AEM	AEM1	0.779	28.144	0.911	0.630	0.882	0.883
	AEM2	0.822	48.850				
	AEM3	0.711	24.204				
	AEM4	0.854	56.127				
	AEM5	0.824	50.362				
	AEM6	0.767	31.156				
RUHS	RUHS1	0.730	18.612	0.869	0.690	0.774	0.798
	RUHS2	0.857	51.440				
	RUHS3	0.896	70.025				
BHB	BHB1	0.904	71.375	0.938	0.792	0.912	0.913
	BHB2	0.891	68.291				
	BHB3	0.879	48.766				
	BHB4	0.885	59.445				

-, means no value.

Supplementary Table S3 shows the standardized factor loadings and cross loadings of the outer model. All the standardized factor loadings for their assigned latent constructs in the shade are greater than their loadings on any other constructs, it indicates that the constructs in this study had good discriminant validity. Supplementary Table S4 shows that all the values of HTMT are between 0.064 and 0.9, no more than 0.9, indicating favorable discriminant validity once again.

### 4.3. Inner model analysis

**Figure 2:**
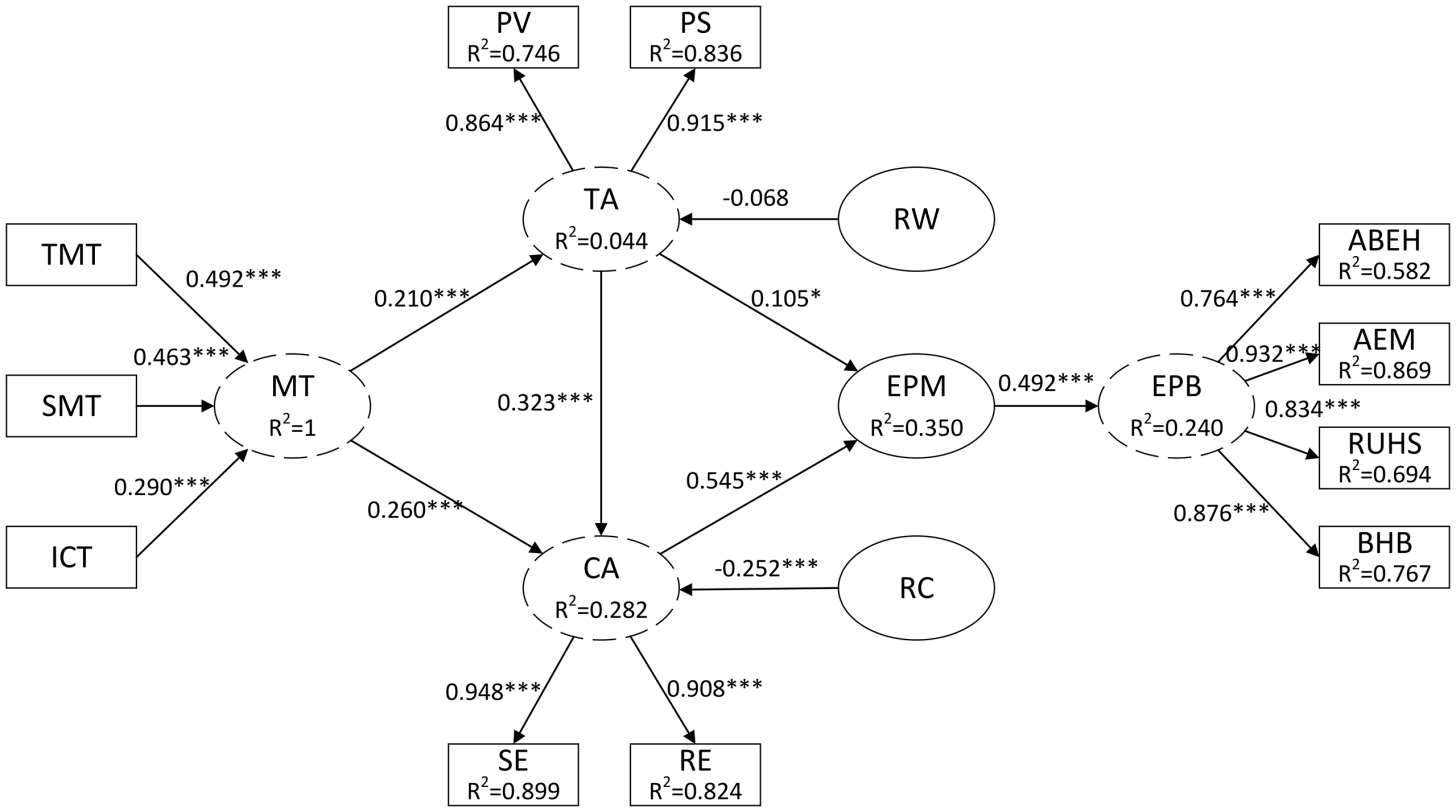
Inner model and path coefficient. 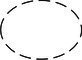 Second-order construct. TMT, Traditional Media Trust; SMT, Social Media Trust; ICT, Interpersonal Communication Trust; MT, Media Trust; TA, Threat Appraisal; CA, Coping Appraisal; PV, Perceived Vulnerability; PS, Perceived Severity; SE, Self-efficacy; RE, Response Efficacy; RW, Rewards; RC, Response Costs; EPM, Epidemic Prevention Motivation; EPB, Epidemic Prevention Behaviors; ABEH, Avoidance Behaviors of Environmental Hazards; AEM, Anti-epidemic Measures; RUHS, Rational Use of Health Services; BHB, Basic Health Behaviors. ^*^p-value < 0.05; ^**^p-value < 0.01; ^***^p-value < 0.001.

In the second step of PLS, the standardized path coefficients, significance, and explanatory power (R^2^) for the inner model are shown in Figure 2. It shows that media trust positively and significantly affects threat appraisal and coping appraisal (H1:β = 0.210, *p*-value < 0.001; H2: β = 0.260, *p*-value < 0.001), rewards negatively but not significantly affect threat appraisal (H3:β = −0.068, *p*-value > 0.05), response costs negatively and significantly affect coping appraisal (H4:β = −0.252, *p*-value < 0.001), threat appraisal and coping appraisal both positively and significantly affect epidemic prevention motivation (H5:β = 0.105, *p*-value < 0.05; H6:β = 0.545, *p*-value < 0.001), threat appraisal positively and significantly affects coping appraisal (H7:β = 0.323, *p*-value < 0.001), and epidemic prevention motivation positively and significantly affects epidemic prevention behaviors (H8:β = 0.492, *p*-value < 0.001). The analysis indicated that RW had no significant direct effect on TA, and thus H3 was not supported, while the remaining seven path hypotheses were significant and supported.

It is also crucial to determine the variance indicated by the R^2^ value in the inner model analysis. The R^2^ value of threat appraisal is 0.044, which indicates that 4.4% of the variance of threat appraisal is explained by media trust. The R^2^ value of coping appraisal is 0.282, which indicates that 28.2% of the variance of coping appraisal is explained by media trust. The R^2^ value of epidemic prevention motivation is 0.350, which indicates that 35.0% variance of epidemic prevention motivation is explained by threat appraisal and coping appraisal. The R^2^ value of epidemic prevention behaviors is 0.240, which indicates that 24.0% of the variance of epidemic prevention behaviors is explained by epidemic prevention motivation. On the whole, the model had acceptable explanatory power.

### 4.4. Multi group analysis

In order to examine the specific group differences by multi group analysis (MGA), a permutation algorithm is necessary to examine MICOM. In this study, permutation *p*-values of step 2 are between 0.2 and 0.9, and are greater than 0.05 but not significant, and several permutation p-values of step 3 are significant, indicating partial measurement invariance. Therefore, as Henseler et al. suggested, it is suitable to perform MGA to determine if structural invariance exists (64).

Table 2 indicates the results of the MGA between the no epidemic experience group and the epidemic experience group. The results showed that in the no epidemic experience group, the path coefficients from RW to TA (β = 0.115, *p*-value > 0.1) and from TA to EPM (β = 0.031, *p*-value >0.1) were not significant, but other path coefficients were. In the epidemic experience group, all the path coefficients were significant. Regarding the comparative study, significant differences were found between the two groups in five paths, including TA to PV (β = 0.089, *p*-value<0.05), MT to CA (β = 0.182, *p*-value < 0.1), CA to RE (β = 0.050, *p*-value < 0.05), CA to SE (β = 0.034, *p*-value < 0.05), and TA to EPM (β = −0.19, *p*-value < 0.1). The personal epidemic experience played a moderating role in these paths. In particular, we found that TA positively and significantly predicted EPM in the epidemic experience group, whereas this path was not significant in the no epidemic experience group, and the difference between the two groups was significant. On the other hand, RW negatively and significantly predicted TA in the epidemic experience group, but this path was not significant in the no epidemic experience group, and the difference between the two groups was not significant either.

**Table 2:** Multi-group analysis result.

Path	Path coefficients original (no epidemic experience group)	Path coefficients original (epidemic experience group)	Path coefficients-diff (no epidemic experience group—epidemic experience group)	*P*-value (no epidemic experience group vs. epidemic experience group)
TMT → MT	0.49***	0.498^***^	−0.008	0.896
SMT → MT	0.445^***^	0.491^***^	−0.046	0.172
ICT → MT	0.299^***^	0.267^***^	0.033	0.414
MT → TA	0.163^*^	0.244^**^	−0.081	0.449
TA → PS	0.926^***^	0.9^***^	0.026	0.193
TA → PV	0.893^***^	0.805^***^	0.089	0.017^*^
RW → TA	0.115	−0.172^*^	0.286	0.205
MT → CA	0.336^***^	0.154^△^	0.182	0.058△
CA → RE	0.929^***^	0.879^***^	0.05	0.022^*^
CA → SE	0.963^***^	0.929^***^	0.034	0.013^*^
RC → CA	−0.296^***^	−0.229^***^	−0.068	0.375
TA → EPM	0.031	0.22^**^	−0.19	0.068^△^
CA → EPM	0.602^***^	0.47^***^	0.132	0.205
TA → CA	0.31^***^	0.35^***^	−0.039	0.679
EPM → EPB	0.479^***^	0.506***	−0.027	0.788
EPB → ABEH	0.749^***^	0.782***	−0.033	0.633
EPB → AEM	0.931^***^	0.933***	−0.003	0.852
EPB → RUHS	0.823^***^	0.843***	−0.02	0.589
EPB → BHB	0.879^***^	0.873***	0.006	0.808

^△^*P*-value < 0.1; ^*^P-value < 0.05; ^**^P-value < 0.01; ^***^p-value < 0.001.

## 5. Discussion

The overall purpose of this study was to explore the influence factors and formation mechanisms of epidemic preventive behaviors implemented by the public. This study conducted empirical research that elucidated the impact of media trust on COVID-19 prevention motivation and behaviors based on the PMT, and evaluated the moderation effect of personal epidemic experience, which focused on the differences in two groups with and without epidemic experience. There are three main findings, as follows.

First, the present study introduced media trust as the information source primarily influencing COVID-19 prevention motivation and behaviors. More specifically, the public acquired information about COVID-19 through various channels, including traditional media, social media, and interpersonal communication. Trust in different information channels was the exogenous variable that produced motivation and behaviors. According to the path coefficients, media trust was positively and significantly related to both threat appraisal and coping appraisal, with a nearly equal degree of correlation to both. Traditional media trust has the strongest relationship with media trust and, further, with protection motivation and behaviors through threat appraisal and coping appraisal. Therefore, in accordance with findings reported by Li et al. (41), traditional media has been evidenced as an important element in COVID-19 prevention and control, which indicates that people rely more on traditional media to obtain authoritative epidemic information, disease dissemination knowledge, and countermeasures, which makes them more likely to develop prevention motivation even in the later stages of the epidemic. However, different from Li et al.’s research that social media trust had a significant effect on coping appraisal but not threat appraisal, social media trust is slightly less but significantly related to media trust so as to positively and significantly effect both threat appraisal and coping appraisal, with a lower degree than traditional media trust in this study. Nowadays, social media have made some progress in enhancing their credibility and competing for the audience market, and have thus become an important channel for people to access information. Although some epidemic-related information is rumor or misleading, people will choose what they personally trust when browsing social media information to learn about an epidemic’s risk. The last one was interpersonal communication trust, which has the lowest path coefficient for people to develop motivation and behaviors since most people believe that the information in personal conversations is partial and inaccurate.

Second, several results of association between PMT constructs in this model are worth discussing. There was a negative but not significant association between rewards and threat appraisal, which was different from the conclusion made by Malak Al-Rasheed (37). The reason for this is that, due to the differences in national situations and cultures, people in China did not strongly perceive the rewards for not taking protective measures. For example, wearing a mask is not taboo in China, so Chinese people are not averse to wearing masks. On the contrary, the negative association between response costs and coping appraisal was significant, indicating that the higher the response costs, the more reluctant individuals are to adopt health protection behaviors. The result concurred with prior findings on PMT (33). Furthermore, it was also confirmed that threat appraisal and coping appraisal were significantly related to epidemic prevention motivation, which explained the 35% variance of epidemic protection motivation in sum. Obviously, coping appraisal had a stronger connection to epidemic prevention motivation than threat appraisal, which shows that the public sometimes generates protection motivation not mainly by fear or dread of disease but mainly by the expectation that adopting the behaviors will produce a good health outcome, which is consistent with the research of Ezati Rad et al. (33). Besides, threat appraisal positively and significantly affected coping appraisal. It indicates that there is a sequence to people’s cognitive assessment process, which includes appraising the current threat first and then the response to it. Last but not least, epidemic prevention motivation also positively and significantly correlated to epidemic prevention behaviors, which contained avoidance behaviors of environmental hazards (ABEH), anti-epidemic measures (AEM), and rational use of health services (RUHS). The connection between epidemic prevention behaviors and anti-epidemic measures was the strongest.

Third, the individuals with and without personal epidemic experience were adopted as different samples to examine the moderation effect of personal epidemic experience on the model by MGA of PLS. In sum, despite the minor variations, the study successfully replicated the PMT model on epidemic prevention behaviors among both the epidemic experience group and the no epidemic experience group, thereby verifying the generalization ability of the model. However, there were also several significant differences between the two groups. The coefficient path from threat appraisal to perceived vulnerability was significant and positive for both groups, but the correlation of the no epidemic experience group was stronger than that of the epidemic experience group. This finding agrees with the perspective of Frewer et al., who hold the view that perceived vulnerability is the subjective cognition of the individual, and that some media provide one-sided information that greatly stimulates the individual’s risk perception and amplifies perceived susceptibility (68). This finding is also consistent with the study of Kahneman and Tversky, which verified that learning and experience with risk affect people’s assessments of risk. When there is a lack of learning and experience with risk, people will overestimate the risk they encounter. However, when they have fully experienced or learned about risk, they will tend to be objective in their evaluation of risk (69). As a result, people without personal epidemic experience are more likely to exaggerate their perceived vulnerability to being infected with COVID-19 due to a lack of direct experience. Moreover, the coefficient paths from media trust to coping appraisal, coping appraisal to response efficacy, and coping appraisal to self-efficacy were significant and positive for both groups, but the correlation of the no epidemic experience group was stronger than that of the other group. This demonstrated that when people have more trust in the information they acquire from the media, people without epidemic experience are more likely to generate coping appraisal, including response efficacy and self-efficacy, than people with epidemic experience. In addition, the coefficient path from threat appraisal to epidemic prevention motivation was not significant in the no epidemic experience group but was significant in the epidemic group, indicating that if people without an epidemic experience just perceive vulnerability and severity, they hardly generate preventive motivation. On the contrary, the more people who had epidemic experiences perceived the appraisal, the more motivated they became. Therefore, personal epidemic experience moderates the model to some extent.

There are both theoretical and practical implications of the findings in the present study. Theoretically, this study made five main contributions. First, it used the PMT to show the entire path of the association from media trust to epidemic prevention behaviors, which had significant implications for understanding how information source and trust influenced behavior. Second, it built four second-order constructs, including media trust, threat appraisal, coping appraisal, and epidemic prevention behaviors. High-order constructs contribute to model simplicity by reducing the number of path model relationships (70). Third, it classified epidemic prevention behaviors into four categories, namely avoidance behaviors of environmental hazards, anti-epidemic measures, rational use of health services, and basic health behaviors, while past studies focused only on one or two specific anti-epidemic behaviors. This study highlighted the influence on other styles of health behaviors except for anti-epidemic behaviors. Fourth, individual epidemic experience played the role of a moderating variable in the model for the first time, since the model was different between the epidemic experience group and the no epidemic experience group. Previous studies, however, used COVID-19 exposure or experiences as exogenous variables (26, 40). Finally, it used the PLS-SEM method, which had rarely been used in previous related studies. PLS algorithm has advantages in handling non-normally distributed data, second-order complex models, and formative constructs.

In practice, these findings indicated that traditional media and social media play an important role in risk management. Public health departments and governments should consider traditional and social media comprehensively to disseminate risk information about public health events. Another important practical implication is that the effect of coping appraisal on motivation formation for COVID-19 prevention could be applied during the COVID-19 pandemic, as most studies have demonstrated that coping appraisal is an effective factor in motivation formation and behavior engagement (39). There is, therefore, a definite need for continued efforts to make efficacy information from traditional media and social media more accessible to generate protection motivation and behaviors. At present, the media use threat information more frequently, but efficacy information is relatively inadequate. To balance the two, the focus should be on efficacy information, using specific prevention behaviors to enhance the public’s evaluation of potential response effects and the ability of individuals to successfully implement recommendations.

This study still has several limitations. First, due to the epidemic prevention and control policy at the time after the spring festival of 2022 in China, when the epidemic had spread to many places, we chose convenient sampling through the Tencent questionnaire platform. As a result, the study may have suffered from selection bias because it did not include the rural poor or people with low educational levels, and the study’s representativeness was limited. Second, media trust, as the one exogenous variable, has insufficient explanatory power for threat appraisal. The result shows that only 4.4% of the variance of threat appraisal is explained by media trust. It indicated that except for media trust, there may be other variables to explain the threat appraisal.

We encourage future research to consider more personal factors such as underlying disease, smoking status, health literacy, and so on that may influence PMT variables and epidemic prevention motivation and behaviors. Furthermore, many other variables influencing threat appraisal should be contained in the model in future studies in order to increase the R^2^ value of threat appraisal and strengthen the explanatory power of the model.

## 6. Conclusion

This study expanded the connotation and extension of PMT and enriched the current literature about the public’s prevention behaviors about COVID-19. Different from past studies, it validated the relationship between public media trust, PMT constructs, and four types of health behaviors in a public health emergency. Three types of media trust, especially traditional media trust and social media trust, were positively and significantly related to both threat appraisal and coping appraisal, which both positively and significantly affect epidemic prevention motivation and behaviors. Moreover, it validated the slightly moderating role of personal epidemic experience in the model for in-depth research. Our findings suggested that traditional and social media were the main channels to disseminate risk information, and public health departments and governments should ensure the accuracy and reliability of their information. Simultaneously, the media should balance threat information and efficacy information in order to generate the public’s prevention motivation and behaviors.

## Data availability statement

The original contributions presented in the study are included in the article/Supplementary material, further inquiries can be directed to the corresponding author.

## Ethics statement

The studies involving human participants were reviewed and approved by Dianchi College of Yunnan University. The participants provided their written informed consent to participate in this study.

## Author contributions

DZ and ZZ: conceptualization. DZ: methodology, software, and formal analysis. ZZ and XM: validation. FS and XM: investigation. FS: resources and data curation. DZ and XM: writing—original draft preparation and funding acquisition. ZZ: writing—review and editing and supervision. XM: visualization. FS: project administration. All authors contributed to the article and approved the submitted version.

## Funding

This research was funded by the Humanities and Social Sciences Research Project of the Education Department of Guizhou Province (2021QN017) and the Science and Technology Program of the Science and Technology Department of Guizhou Province (qiankehejichu-ZK[2022]general 375).

## Conflict of interest

The authors declare that the research was conducted in the absence of any commercial or financial relationships that could be construed as a potential conflict of interest.

## Publisher’s note

All claims expressed in this article are solely those of the authors and do not necessarily represent those of their affiliated organizations, or those of the publisher, the editors and the reviewers. Any product that may be evaluated in this article, or claim that may be made by its manufacturer, is not guaranteed or endorsed by the publisher.
