# Supplementary material for: Impact of media trust and personal epidemic experience on epidemic prevention behaviors in the context of COVID-19: A cross-sectional study based on protection motivation theory
Source: Front Public Health. 2023 Apr 13;11:1137692. doi: 10.3389/fpubh.2023.1137692 (PMC10133695; doi:10.3389/fpubh.2023.1137692)
Supplement: Supplementary file 2 [file Data_Sheet_2.docx]

Supplementary Material

Impact of media trust and personal epidemic experience on epidemic prevention behaviors in the context of COVID-19: A cross-sectional study based on Protection Motivation Theory

Dan Zhang, Fan Su, Xiaoxia Meng, Zhixin Zhang*

*** Correspondence:** Zhixin Zhang: zhangzhixin022@hotmail.com

# Supplementary Data

Supplementary Material should be uploaded separately on submission. Please include any supplementary data, figures and/or tables.

Supplementary material is not typeset so please ensure that all information is clearly presented, the appropriate caption is included in the file and not in the manuscript, and that the style conforms to the rest of the article.

# Supplementary Figures and Tables

For more information on Supplementary Material and for details on the different file types accepted, please see [here](https://www.frontiersin.org/guidelines/author-guidelines#supplementary-material).

**Table S1. Studies on health protective behaviors of COVID-19.**

| Authors | Health Protective Behaviors |
| --- | --- |
| Kowalski & Black (2021) [32] | Hand washing, hand disinfection, social distancing, self-isolation, object disinfection, and mask wearing behaviors |
| Ezati & Mohseni et al. (2021) [33] | Go out of the house only in emergencies; avoid kissing or shaking hands; avoid touching the mouth, nose, and eyes; keep a 1–2m social distance; use tissue while coughing/sneezing; avoid crowded places; wash hands regularly with water and soap for at least 20 s; avoid unnecessary travel; stress management; wear masks while approaching suspected cases. |
| Park (2021) [34] | There are 14 items, including washing hands more frequently; using hand sanitizers; covering nose and mouth when coughing or sneezing; avoiding touching your eyes, nose, and mouth; avoiding using public transportation; avoiding going out; avoiding crowded places; cancelling social activities; avoiding physical contact et al. |
| Yıldırım & Güler (2020) [35] | There are 16 items, including wearing a mask; avoiding travelling on subways or buses; avoiding large gatherings of people; avoiding travelling to COVID-19-infected areas; taking an herbal supplement; eating a balanced diet; using disinfectants; making sure to get sufficient sleep; paying more attention to cleanliness; exercising regularly et al. |
| Prasetyo & Castillo et al. (2020) [36] | Practice proper handwashing to prevent the spread of the virus; use hand sanitizer more often; always wash hands when going outside; practice 1-meter social distancing; the company/school implements work from home; always wear a face mask when going outside; properly dispose of the face mask. |

**Table S2.** **Items used to measure research constructs**

| Second-order constructs | Constructs | Source | Indicator code | Items |
| --- | --- | --- | --- | --- |
| Media trust | Traditional media trust | Adapted from Li & Sun (2021) [41] | TMT1 | The degree of trust in epidemic-related information released by national and local TV news, radio, and newspapers. |
|  |  |  | TMT2 | The degree of trust in epidemic-related information from interview with medical experts. |
|  |  |  | TMT3 | The degree of trust in epidemic-related information released by government websites. |
|  | Social media trust | Adapted from Li & Sun (2021) | SMT1 | The degree of trust in epidemic-related information released by the official Weibo, WeChat, and Tiktok accounts of influential self-media operators. |
|  |  |  | SMT2 | The degree of trust in epidemic-related information released by the self-media of knowledge-based internet celebrities such as practicing physicians and university professors. |
|  |  |  | SMT3 | The degree of trust in epidemic-related information released by medical science platforms such as Ding Xiang Yi Sheng. |
|  | Interpersonal communication trust | Adapted from Li & Sun (2021) | ICT1 | The degree of trust in epidemic-related information released by WeChat groups or WeChat Moments. |
|  |  |  | ICT2 | The degree of trust in epidemic-related information obtained from conversations with friends, relatives, and neighbors. |
| Threat appraisal | Perceived vulnerability | Adapted from Al-Rasheed (2020) [37] | PV1 | Being in a crowded place can infect me with COVID-19. |
|  |  |  | PV2 | I would get COVID-19 if there are COVID-19 infected persons around when I am shopping. |
|  |  |  | PV3 | Without proper protection, I am susceptible to COVID-19. |
|  | Perceived severity | Adapted from Prasetyo & Castillo et al. (2020) [36] | PS1 | I think COVID-19 is a very serious disease. |
|  |  |  | PS2 | If one is infected with COVID-19, it may lead to death. |
|  |  |  | PS3 | I know that the sequela of COVID-19 is very serious. |
|  |  |  | PS4 | If I get infected with COVID-19, my family will suffer. |
|  | Rewards | Adapted from Al-Rasheed (2020) | RW1 | Shopping without wearing a mask makes me feel more convenient. |
|  |  |  | RW2 | During the epidemic closure, I was happy to get together with my family and friends. |
|  |  |  | RW3 | Using masks more than once saves me money and saves masks for society. |
|  |  |  | RW4 | Shorter hand washing time saves water and reduces costs. |
|  |  |  | RW5 | Keeping family gatherings can strengthen unity. |
| Coping appraisal | Response efficacy | Adapted from Al-Rasheed (2020) | RE1 | Vaccination can significantly reduce my risk of COVID-19 infection. |
|  |  |  | RE2 | Using masks and gloves and keeping a social distance can protect me from COVID-19. |
|  |  |  | RE3 | Staying at home and avoiding gatherings can protect me from contracting COVID-19. |
|  | Self-efficacy | Adapted from Al-Rasheed (2020) | SE1 | I believe I can avoid contracting COVID-19 by taking good precautions. |
|  |  |  | SE2 | I can adhere strictly to the outbreak protection recommendations. |
|  |  |  | SE3 | I know exactly how to use masks and disinfectants. |
|  |  |  | SE4 | I can control myself to stay at home as much as possible unless there is an emergency. |
|  | Response cost | Adapted from Al-Rasheed (2020) | RC1 | If I refuse to go out with or visit friends and family, they may make fun of me. |
|  |  |  | RC2 | I am reluctant to use masks and disinfectants because it takes too much time. |
|  |  |  | RC3 | Using masks and disinfectants is costly. |
|  | Epidemic prevention motivation | Adapted from Al-Rasheed (2020) | EPM | I am willing to continue following all protective measures to prevent COVID-19. |
| Epidemic prevention behaviors | Avoidance behavior of environmental hazards | Adapted from Yıldırım & Güler. (2020) [35] | ABEH1 | I try to avoid taking public transportation such as subways, buses, trains, and planes. |
|  |  |  | ABEH2 | I try to avoid crowded places (e.g., restaurants, shopping malls, etc.). |
|  |  |  | ABEH3 | I try to avoid visiting medium and high-risk areas. |
|  |  |  | ABEH4 | To prevent the spread of the virus, I try not to visit relatives or friends’ homes. |
|  | Anti-epidemic measures | Adapted from Ezati & Mohseni et al. (2021) [33] | AEM1 | I try to avoid physical contact with others (e.g., shaking hands, hugging, etc.) and to maintain a social distance of more than 1 meter. |
|  |  |  | AEM2 | I often wash my hands with hand sanitizer for at least 20 seconds. |
|  |  |  | AEM3 | I always wear a mask when I go out during the epidemic. |
|  |  |  | AEM4 | I try to avoid touching my eyes, mouth, and nose with my hands. |
|  |  |  | AEM5 | I always use disinfectant to disinfect my items. |
|  |  |  | AEM6 | When I cough or sneeze, I consciously use a tissue, handkerchief, or elbow to cover my mouth and nose. |
|  | Rational use of health services | Adapted from Yıldırım & Güler. (2020) | RUHS1 | I am fully vaccinated against COVID-19. |
|  |  |  | RUHS2 | I pay more attention to my health and have regular physical examinations. |
|  |  |  | RUHS3 | I get medical treatment in time after I get sick and follow the doctor’s advice. |
|  | Basic health behaviors | Adapted from Yıldırım & Güler. (2020) | BHB1 | I pay more attention to maintaining a healthy, nutritious, and balanced diet. |
|  |  |  | BHB2 | I pay more attention to ensuring sufficient sleep. |
|  |  |  | BHB3 | I pay more attention to keeping the surrounding environment clean. |
|  |  |  | BHB4 | I pay more attention to exercising and improving immunity. |

**Table S3.** **Standardized factor loadings and cross loadings of the outer model**

|  | TMT | SMT | ICT | PV | PS | RW | RE | SE | RC | EPM | ABEH | AEM | RUHS | BHB |
| --- | --- | --- | --- | --- | --- | --- | --- | --- | --- | --- | --- | --- | --- | --- |
| TMT1 | 0.865 | 0.405 | 0.26 | 0.256 | 0.219 | -0.141 | 0.305 | 0.357 | -0.154 | 0.329 | 0.277 | 0.306 | 0.302 | 0.306 |
| TMT2 | 0.87 | 0.486 | 0.297 | 0.185 | 0.137 | -0.132 | 0.25 | 0.322 | -0.16 | 0.221 | 0.297 | 0.334 | 0.325 | 0.351 |
| TMT3 | 0.838 | 0.346 | 0.177 | 0.241 | 0.22 | -0.157 | 0.25 | 0.34 | -0.181 | 0.344 | 0.267 | 0.286 | 0.317 | 0.265 |
| SMT1 | 0.335 | 0.769 | 0.529 | 0.057 | -0.03 | 0.173 | 0.048 | 0.11 | 0.139 | 0.023 | 0.096 | 0.155 | 0.187 | 0.215 |
| SMT2 | 0.463 | 0.893 | 0.51 | 0.11 | 0.108 | 0.03 | 0.204 | 0.247 | -0.023 | 0.197 | 0.2 | 0.325 | 0.34 | 0.352 |
| SMT3 | 0.417 | 0.854 | 0.432 | 0.079 | 0.1 | 0.067 | 0.202 | 0.236 | -0.021 | 0.168 | 0.131 | 0.227 | 0.291 | 0.306 |
| ICT1 | 0.272 | 0.538 | 0.921 | 0.11 | 0.08 | 0.199 | 0.08 | 0.106 | 0.159 | 0.085 | 0.097 | 0.149 | 0.132 | 0.158 |
| ICT2 | 0.259 | 0.533 | 0.918 | 0.079 | 0.056 | 0.163 | 0.09 | 0.143 | 0.16 | 0.111 | 0.085 | 0.148 | 0.163 | 0.203 |
| PV1 | 0.162 | 0.048 | 0.115 | 0.841 | 0.458 | -0.017 | 0.225 | 0.218 | 0.034 | 0.165 | 0.271 | 0.212 | 0.178 | 0.115 |
| PV2 | 0.18 | 0.101 | 0.08 | 0.845 | 0.514 | -0.028 | 0.184 | 0.201 | -0.015 | 0.167 | 0.264 | 0.231 | 0.188 | 0.182 |
| PV3 | 0.315 | 0.098 | 0.063 | 0.81 | 0.491 | -0.088 | 0.266 | 0.344 | -0.118 | 0.308 | 0.335 | 0.263 | 0.249 | 0.16 |
| PS1 | 0.305 | 0.147 | 0.124 | 0.46 | 0.781 | -0.13 | 0.352 | 0.354 | -0.103 | 0.278 | 0.262 | 0.301 | 0.296 | 0.254 |
| PS2 | 0.133 | 0.045 | 0.057 | 0.453 | 0.815 | -0.04 | 0.215 | 0.278 | -0.084 | 0.264 | 0.182 | 0.213 | 0.213 | 0.226 |
| PS3 | 0.185 | 0.041 | 0.048 | 0.517 | 0.83 | -0.004 | 0.258 | 0.288 | -0.049 | 0.237 | 0.172 | 0.17 | 0.184 | 0.138 |
| PS4 | 0.067 | 0.002 | 0 | 0.418 | 0.728 | -0.006 | 0.254 | 0.236 | -0.05 | 0.188 | 0.21 | 0.183 | 0.209 | 0.136 |
| RW1 | -0.17 | 0.066 | 0.129 | -0.028 | -0.01 | 0.727 | -0.15 | -0.186 | 0.612 | -0.186 | -0.252 | -0.288 | -0.2 | -0.191 |
| RW2 | -0.1 | 0.154 | 0.174 | -0.046 | -0.03 | 0.791 | -0.09 | -0.141 | 0.457 | -0.149 | -0.19 | -0.175 | -0.11 | -0.101 |
| RW3 | -0.14 | 0.093 | 0.188 | -0.034 | -0.04 | 0.816 | -0.18 | -0.197 | 0.661 | -0.185 | -0.243 | -0.192 | -0.18 | -0.129 |
| RW4 | -0.16 | 0.051 | 0.217 | 0.016 | -0.02 | 0.78 | -0.23 | -0.231 | 0.685 | -0.267 | -0.186 | -0.244 | -0.21 | -0.189 |
| RW5 | -0.15 | 0.052 | 0.149 | -0.059 | -0.07 | 0.896 | -0.13 | -0.197 | 0.538 | -0.159 | -0.311 | -0.273 | -0.15 | -0.126 |
| RE1 | 0.296 | 0.158 | 0.035 | 0.221 | 0.257 | -0.101 | 0.82 | 0.539 | -0.147 | 0.368 | 0.34 | 0.314 | 0.347 | 0.295 |
| RE2 | 0.3 | 0.168 | 0.115 | 0.21 | 0.263 | -0.159 | 0.904 | 0.674 | -0.213 | 0.42 | 0.345 | 0.392 | 0.367 | 0.358 |
| RE3 | 0.206 | 0.147 | 0.081 | 0.262 | 0.355 | -0.163 | 0.836 | 0.644 | -0.217 | 0.383 | 0.397 | 0.423 | 0.388 | 0.363 |
| SE1 | 0.265 | 0.184 | 0.107 | 0.212 | 0.222 | -0.116 | 0.711 | 0.796 | -0.198 | 0.47 | 0.4 | 0.46 | 0.411 | 0.399 |
| SE2 | 0.378 | 0.186 | 0.113 | 0.307 | 0.365 | -0.219 | 0.575 | 0.872 | -0.282 | 0.607 | 0.459 | 0.513 | 0.487 | 0.457 |
| SE3 | 0.335 | 0.228 | 0.109 | 0.219 | 0.245 | -0.173 | 0.583 | 0.826 | -0.232 | 0.467 | 0.411 | 0.547 | 0.512 | 0.501 |
| SE4 | 0.314 | 0.183 | 0.114 | 0.259 | 0.376 | -0.224 | 0.494 | 0.768 | -0.248 | 0.446 | 0.528 | 0.536 | 0.409 | 0.38 |
| RC1 | -0.14 | 0.036 | 0.158 | -0.066 | -0.06 | 0.523 | -0.12 | -0.207 | 0.774 | -0.232 | -0.165 | -0.149 | -0.16 | -0.116 |
| RC2 | -0.19 | 0.018 | 0.163 | -0.069 | -0.11 | 0.641 | -0.24 | -0.31 | 0.91 | -0.289 | -0.28 | -0.333 | -0.27 | -0.263 |
| RC3 | -0.15 | 0.033 | 0.115 | 0.048 | -0.04 | 0.521 | -0.18 | -0.197 | 0.83 | -0.263 | -0.146 | -0.175 | -0.23 | -0.22 |
| EPM1 | 0.343 | 0.159 | 0.106 | 0.256 | 0.307 | -0.204 | 0.458 | 0.611 | -0.313 | 1 | 0.428 | 0.449 | 0.459 | 0.367 |
| ABEH1 | 0.225 | 0.111 | 0.06 | 0.315 | 0.197 | -0.241 | 0.365 | 0.402 | -0.194 | 0.316 | 0.788 | 0.47 | 0.31 | 0.287 |
| ABEH2 | 0.281 | 0.17 | 0.102 | 0.31 | 0.187 | -0.258 | 0.343 | 0.493 | -0.187 | 0.315 | 0.887 | 0.629 | 0.377 | 0.423 |
| ABEH3 | 0.281 | 0.089 | 0.013 | 0.233 | 0.228 | -0.23 | 0.344 | 0.393 | -0.23 | 0.398 | 0.712 | 0.472 | 0.457 | 0.376 |
| ABEH4 | 0.278 | 0.179 | 0.132 | 0.281 | 0.237 | -0.277 | 0.333 | 0.483 | -0.199 | 0.37 | 0.862 | 0.677 | 0.393 | 0.456 |
| AEM1 | 0.293 | 0.189 | 0.141 | 0.324 | 0.262 | -0.236 | 0.372 | 0.48 | -0.202 | 0.417 | 0.688 | 0.779 | 0.481 | 0.48 |
| AEM2 | 0.29 | 0.241 | 0.161 | 0.214 | 0.197 | -0.211 | 0.318 | 0.446 | -0.196 | 0.273 | 0.512 | 0.822 | 0.546 | 0.626 |
| AEM3 | 0.297 | 0.175 | 0.044 | 0.254 | 0.257 | -0.295 | 0.399 | 0.562 | -0.323 | 0.461 | 0.609 | 0.711 | 0.585 | 0.508 |
| AEM4 | 0.276 | 0.262 | 0.18 | 0.213 | 0.183 | -0.227 | 0.329 | 0.478 | -0.205 | 0.301 | 0.551 | 0.854 | 0.54 | 0.626 |
| AEM5 | 0.246 | 0.293 | 0.157 | 0.171 | 0.181 | -0.156 | 0.308 | 0.499 | -0.164 | 0.305 | 0.461 | 0.824 | 0.534 | 0.636 |
| AEM6 | 0.321 | 0.194 | 0.081 | 0.175 | 0.232 | -0.217 | 0.388 | 0.53 | -0.245 | 0.393 | 0.518 | 0.767 | 0.563 | 0.565 |
| RUHS1 | 0.301 | 0.189 | 0.066 | 0.178 | 0.241 | -0.202 | 0.327 | 0.441 | -0.191 | 0.403 | 0.394 | 0.454 | 0.73 | 0.43 |
| RUHS2 | 0.288 | 0.301 | 0.149 | 0.2 | 0.219 | -0.124 | 0.307 | 0.436 | -0.228 | 0.335 | 0.358 | 0.609 | 0.857 | 0.707 |
| RUHS3 | 0.329 | 0.314 | 0.171 | 0.232 | 0.255 | -0.148 | 0.435 | 0.514 | -0.242 | 0.416 | 0.429 | 0.62 | 0.896 | 0.748 |
| BHB1 | 0.338 | 0.348 | 0.171 | 0.18 | 0.227 | -0.142 | 0.352 | 0.47 | -0.249 | 0.249 | 0.394 | 0.63 | 0.741 | 0.904 |
| BHB2 | 0.306 | 0.322 | 0.216 | 0.134 | 0.2 | -0.092 | 0.346 | 0.417 | -0.19 | 0.298 | 0.378 | 0.614 | 0.658 | 0.891 |
| BHB3 | 0.365 | 0.292 | 0.119 | 0.194 | 0.226 | -0.184 | 0.402 | 0.542 | -0.259 | 0.452 | 0.491 | 0.689 | 0.733 | 0.879 |
| BHB4 | 0.271 | 0.285 | 0.197 | 0.142 | 0.196 | -0.138 | 0.312 | 0.463 | -0.181 | 0.3 | 0.44 | 0.641 | 0.613 | 0.885 |

Note: The shaded part represents the standardized factor loadings.

**Table S4.** **Results of discriminant validity by HTMT**

|  | ICT | TMT | BHB | RC | RE | RUHS | RW | PS | PV | AEM | SMT | SE | ABEH | EPM |
| --- | --- | --- | --- | --- | --- | --- | --- | --- | --- | --- | --- | --- | --- | --- |
| ICT | - | - | - | - | - | - | - | - | - | - | - | - | - | - |
| TMT | 0.35 | - | - | - | - | - | - | - | - | - | - | - | - | - |
| BHB | 0.23 | 0.413 | - | - | - | - | - | - | - | - | - | - | - | - |
| RC | 0.22 | 0.233 | 0.277 | - | - | - | - | - | - | - | - | - | - | - |
| RE | 0.11 | 0.384 | 0.459 | 0.268 | - | - | - | - | - | - | - | - | - | - |
| RUHS | 0.2 | 0.464 | 0.9 | 0.33 | 0.542 | - | - | - | - | - | - | - | - | - |
| RW | 0.25 | 0.209 | 0.199 | 0.852 | 0.225 | 0.255 | - | - | - | - | - | - | - | - |
| PS | 0.1 | 0.276 | 0.28 | 0.106 | 0.425 | 0.367 | 0.064 | - | - | - | - | - | - | - |
| PV | 0.13 | 0.332 | 0.216 | 0.107 | 0.341 | 0.317 | 0.064 | 0.743 | - | - | - | - | - | - |
| AEM | 0.19 | 0.424 | 0.806 | 0.313 | 0.523 | 0.82 | 0.326 | 0.33 | 0.343 | - | - | - | - | - |
| SMT | 0.73 | 0.594 | 0.409 | 0.095 | 0.225 | 0.408 | 0.131 | 0.143 | 0.124 | 0.336 | - | - | - | - |
| SE | 0.17 | 0.48 | 0.609 | 0.349 | 0.877 | 0.697 | 0.274 | 0.454 | 0.381 | 0.738 | 0.29 | - | - | - |
| ABEH | 0.12 | 0.396 | 0.544 | 0.292 | 0.52 | 0.594 | 0.338 | 0.323 | 0.437 | 0.812 | 0.205 | 0.66 | - | - |
| EPM | 0.12 | 0.383 | 0.382 | 0.348 | 0.507 | 0.528 | 0.246 | 0.343 | 0.291 | 0.481 | 0.174 | 0.669 | 0.473 | - |
